# Third-party imitation is not restricted to humans

**DOI:** 10.1038/s41598-025-11665-9

**Published:** 2025-09-04

**Authors:** Esha Haldar, Ariana Hernández Sánchez, Claudio Tennie, Sara Torres Ortiz, Janneke Vos, Maurice Valbert, Auguste M. P. von Bayern

**Affiliations:** 1 Max-Planck-Institute for Biological Intelligence (Seewiesen), Starnberg, Germany; 2 Ludwig-Maximilians-Universität München, Munich, Germany; 3 Comparative Cognition Research Station, Loro Parque Fundacion, Tenerife, Spain; 4 University of Tübingen, Tübingen, Germany; 5 University of South Denmark, Kerteminde, Denmark; 6 Wageningen University & Research, Wageningen, Netherlands

**Keywords:** Third-party imitation, Third-party learning, Animal culture, Parrot imitation, Intransitive action imitation, Evolution, Zoology

## Abstract

Imitation of cultural practices is ubiquitous in humans and often involves faithful copying of intransitive (i.e., non-object directed) gestures and societal norms which play a crucial role in human cumulative cultural evolution. Apart from learning these directly from a tutor, humans often learn passively as third-party observers from the interactions of two or more individuals. Whether third-party imitation has evolved outside humans remains unknown. In the current study, we investigated whether undomesticated blue-throated macaws (*Ara glaucogularis*) could imitate in a third-party setting. A naïve test group (*N* = 6) passively observed a conspecific demonstrator performing rare intransitive actions in response to specific human gestural commands. Directly afterwards, the observer received the same gestural commands and performance-contingent rewards. An equally naïve control group (*N* = 5) was tested correspondingly, in the absence of third-party demonstrations. The test group learned more target actions (mean = 4.16 versus mean = 2.2) in response to the specific commands, significantly faster and performed them more accurately than the control group. The test group also spontaneously imitated some of the actions even before they received any gestural commands or rewards. Our findings show that third-party imitation, even for intransitive actions, exists outside humans, allowing for rapid adaption to group specific behaviours and possibly cultural conventions in parrots.

**Supplementary Information:**

The online version contains supplementary material available at 10.1038/s41598-025-11665-9.

## Introduction

Human cumulative culture is largely underpinned by the human ability to imitate^1^ along with other social learning processes (e.g., emulation) and pedagogy, that enable faithful transmission of cultural knowledge through generations. Heyes defined imitation as when an observer “learns and reproduces ‘something’ about the topography of the model’s body movements ”^2^ whereas in other kinds of social learning, an observer learns about the goal of action (*goal emulation*)^3^ or location and characteristics of objects (*local and stimulus enhancement*)^4^. Human children develop the tendency to imitate an adult who is directly demonstrating an action to the child (‘second-person imitation’^5^-A learns from B), from the first year of their life^6^. They also develop third-party imitation (A learns from B and C interacting) ability only towards the end of their second year^6–9^ perhaps linked to the development of an understanding for self-other equivalence^7,10^. Third-party imitation entails the replication of an *act* in the appropriate interactive context after passively observing the act in the interaction of two individuals^5,6^. Thus, in third-party imitation, the observer can also acquire knowledge (e.g. normative knowledge) about the interaction itself, i.e. how to behave in a certain interactive situation. For example, in some hunter-gatherer societies, the socially appropriate conduct expected of a particular gender in specific situations is acquired through passive observational learning^11,12^. Thus, third-party imitation is an important skill that has been reported only in humans^5^ and the evolution of this ability remains largely unexplored. A comparative approach investigating third-party imitation in distantly and non-distantly related non-human species promises to shed light on whether this ability evolved in other animal taxa and its mechanistic underpinnings.

Imitation of a single demonstrator (‘second-person’ or ‘second-party’ imitation’)^5^ has been reported in a few non-human species, e.g., in two-target task studies in which observers show significantly more target observed acts than non-observed ones^13–16^. Apart from whether imitation happens in second-party or third-party settings, it is further distinguished between whether the copied body movements or actions of the model are already present within the behavioural repertoire of the species, which is referred to as *contextual imitation*^17^ or whether the imitated actions are completely novel, which is called *production learning*^17^ or (-by other scientists-) “*true imitation*^18^. Thus, third-party imitation can be *contextual* (actions are familiar but not produced in an interactive context before) or focus on *production learning* (novel or improbable actions), with actions demonstrated through the interaction of two individuals in the presence of a passive observer.

Considering that imitation studies involving object-directed-actions (e.g., two-target task studies^13–16^) often cannot exclude alternative social learning mechanisms such as goal emulation, or stimulus enhancement, evidence for true or contextual imitation can be obtained most convincingly in studies that focus on intransitive actions, i.e. actions that do not involve objects or goals^19^. Here, only the topography of body movements can be the target of social learning^19^. Animal studies of imitation of intransitive actions remain rare^20–25^ and restricted to second-party settings. Maybe surprisingly, given that gestural communication is ubiquitous in non-human primate species^26,27^, gestures—often in the form of intransitive actions—are typically either biologically predisposed responses^28^ or ritualized interactions^29,30,^ but do not seem to be acquired through imitation^29,31^ (neither through second-party nor third-party imitation). Concerning imitation of intransitive actions, Moore^32^ reported ‘second-party’ imitation of intransitive gestures in an African grey parrot (*Psittacus erithacus*). The experimenters interacted directly with the parrot several times a day, performing certain stereotyped body movements. Nevertheless, over the course of five years, this parrot produced 10 actions using head, beak, wings and feet and was claimed to have imitated intransitive actions of the human demonstrators. The other few studies that have reported imitation of intransitive actions in animals have used the “Do-as-I-do” (DAID) or “Do-as-the-other-does” (DATOD) paradigm, in which the animals are actively trained by the experimenter to pay attention to and copy either a human or a conspecific demonstrator^20–25^. All these studies were conducted in a second-party setting (a single model demonstrated the actions) and thus did not address third-party imitation.

Third-party imitation, i.e., imitation of a demonstrator interacting with another individual, has been systematically tested in human children. Here the subjects passively observed an interaction between two individuals^6,7^ and could reproduce the relevant action when tested in the same interactive context. However, in non-human animals, only one single study has tested third-party imitation, namely a study on dogs (*Canis familiaris)*, a domesticated species^5^. Here, a trained dog performed one of two intransitive actions in response to verbal commands of an experimenter, while a task-naïve, observer dog (i.e., the “third-party”) watched the interaction. Subsequently, it was tested whether the observer dog would show the actions demonstrated by the trained conspecific using the same verbal commands. The dogs failed to show evidence of third-party imitation in this study. Yet, even if some dogs had been successful, the obtained results could have been confounded by domestication effects in dogs^5^. Tennie and colleagues^33^ had tested unenculturated chimpanzees for *contextual imitation* in a comparable third-party setting, where a trained chimpanzee performed an arbitrary action to obtain food from an experimenter (social context) while the naïve observers watched the interaction. Except for one chimpanzee, the rest failed to imitate the arbitrary action in this study, consistent with previous findings in the species, where naïve chimpanzees were unable to reproduce novel begging gestures (*production learning*) demonstrated by a model to obtain food from an experimenter^31^. In a different set of studies, Pepperberg and colleagues, used a method called “model/rival technique”^34^ in which two trainers interacted with each other while a single African grey parrot observed their interaction^35^. However, this technique was employed as a general tool for training the parrot to learn object labels by vocal imitation rather than for specifically investigating its ability to learn via third-party imitation. Moreover, the grey parrot received direct interactive training concurrently and hence the effect of learning as a third party cannot be teased apart retrospectively from these experiments. Thus, third-party imitation in non-human and non-domesticated animals has not been studied specifically, even though it is likely to exist, and a comparative approach promises insights into the evolution of third-party imitation.

We hypothesize, that third-party imitation should most likely prevail in social animals in which social behavioural repertoire (including those mediated through vocal^36,37^ and gestural^38,39^ communication) varies within and across populations^40–42^. For example, third-party imitation may be observed in animal societies with fission-fusion dynamics which brings about frequent changes in group composition^43^ or in matrilocal/patrilocal societies, where one sex of the species disperses to join unrelated groups^44^. Here, cultural learning about group-typical social behaviours^40^ including idiosyncratic behaviours^45^ might aid group cohesion and some might be required for integration into a group^45^.

In this study, we aim to test third-party imitation in parrots, focusing on *contextual* imitation of intransitive actions, i.e., imitation of actions that are present in the repertoire of the species but occur infrequently and demonstrated through the interaction between a human experimenter and a conspecific. As indicated by the aforementioned studies^32,35^ parrots are particularly interesting candidates for studying third-party imitation. Parrots characteristically live in fission-fusion societies^46^ in which learning group-typical interactive social behaviours, such as gestures or coordinated (synchronised) movements by social observation, may be important for group cohesion. Parrots are among the very few animal taxa possessing vocal imitation ability^47–49^ tool-making skills^50,51^ foraging cultures^52^ and vocal dialects^36^. They have been also known to show gestural communication^53,54^. Perhaps most importantly, parrots have previously been successful in second-party imitation of transitive and intransitive actions^32,55,56^ and they appear to learn from a human third party- setting as a previous study suggests^35^ but it remains untested whether they are capable of learning via third-party imitation.

To convincingly investigate a non-human species’ capacity for third-party imitation, it is essential to ensure the following conditions: (1) demonstrations must not be overtly directed at the subjects using human-specific cues (e.g., ostensive signals like calling or eye gaze), to ensure passive observation; (2) subjects should not be human-enculturated (as with domestic dogs), in order to maintain ecological validity; and most importantly (3) test subjects (observers) must have no prior training in either the target actions or imitation/copying actions itself (as seen in DAID paradigms^20–25^), to avoid confounding effects of automatic imitation (sensorimotor experience gained through trained actions) or second-party imitation (if DAID trained, animals may have a predisposition to copy actions and ignore the interactive context).

**Fig. 1:**
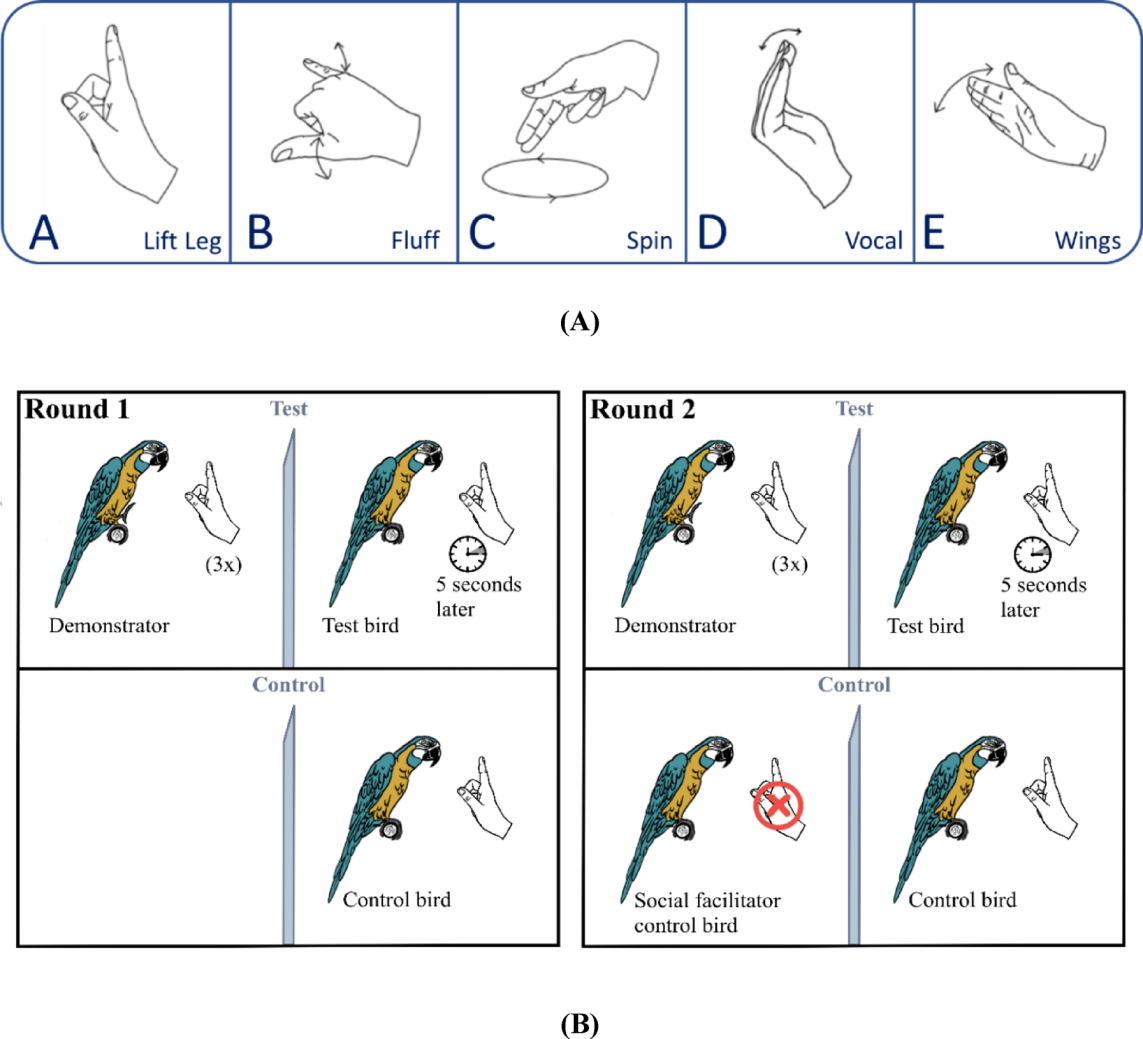
Illustration of the experimental procedure. **A**. The gestural commands associated with the target actions. **B.** The left panel shows the first round of testing, with the demonstrator and the test subject in the test group (left up) and the control subject tested alone in the control group (left down). The demonstrator performs an action thrice in response to the gestural command of the experimenter, 3–5 s following which the second experimenter gives the same gestural command to the test subject in a test trial. The control subject is only given the gestural command without any demonstration. In round 2, shown on the right panel, the testing contingency remains the same except for the addition of a social facilitator bird in the adjacent room of the control subject, who sits on the perch passively without performing any action.

We complied with all points raised above and operationally defined third-party imitation as copying rare target actions, matching the model’s action topography upon observing them in a specific interactive social context, i.e., after observing the behavioural response of a conspecific demonstrator to a human gesture (here). To ensure the actions used to test for contextual imitation (rather than ‘true imitation’ or ‘production learning’) were sufficiently rare, we established a baseline by recording the natural frequency/occurrence of the target actions and through post-hoc analysis. In the experiment, a test group of (*N* = 6) hand-raised blue-throated macaws that were habituated to receiving food from human hands, but were not trained to perform specific behaviours on command and were never intentionally trained to imitate, observed a trained conspecific demonstrator performing five intransitive target actions (*lift leg*,* fluff*,* spin*,* vocal* and *flap wings*), one at a time, in a session. The order of actions demonstrated was randomised across subjects. Each target action was performed in response to specific hand commands from the experimenter (see Fig. 1A). Subjects in the test group observed these demonstrations passively from an adjacent room separated by a transparent plexiglass (see Fig. 1B). Following these demonstrations (i.e., after 3–5 s), a second experimenter (who had been passively present in the adjacent room together with the subject) gave the same gestural commands to the subject (see Supplementary Videos S1-5), testing whether subjects would express the demonstrated target actions within a timeframe of 12 (± 2) seconds of response time, standardized arbitrarily to match the assumed attention span of a macaw (based on observation of experimenters). A control group (*N* = 5) was exposed to the same gestural commands, yet without ever observing any target action linked to the commands (see Supplementary Video S6-7). We rewarded the subjects in both test and control groups for performing the target action with the respective command. Overall, this study aimed to investigate whether macaws learned to reproduce intransitive target actions in the correct interactive context (response to correct gestural command) more accurately and faster, after observing a model perform them in a third-party setting than in the absence of demonstrations.

## Results

### Baseline: establishing natural occurrence rates of target actions

A baseline of the natural occurrence of the chosen intransitive actions *‘lift leg’ ‘fluff’*,* ‘spin’*,* ‘vocal’* and *‘flap wings’* was established by conducting 4 hours of focal behavioural sampling of 11 subjects (44 hours in total) (one control was unavailable, more details in methods) in the aviaries before the onset of the experiment. Focal sampling throughout 15 min per subject were conducted for 16 days (resulting in a total of 4 h in total per individual). None of the five focal behaviours were exhibited as part of social interaction, nor were they used as display behaviour for communication. From the yielded cumulative scores for each action (see Supplementary Information), we obtained the rate at which they occur in 12 sec response interval we gave to our subjects in the test and control conditions (see above). The actions were considered very improbable to occur naturally (according to criteria set post-hoc) within the response time except *‘vocal’* occurring infrequently (5%). The rate at which the other actions occurred naturally: *‘fluff’*: 0.5%; *‘flap wings’*: 0.04%; *‘spin’*: 0.001% and *‘lift leg’* 0%. *Lift leg* never occurred unless the bird scratched, nibbled its toes or fended of an aggressive approach by holding out a leg (which happened 0.03%) (see SM for details).

### Number of actions learnt

**Fig. 2:**
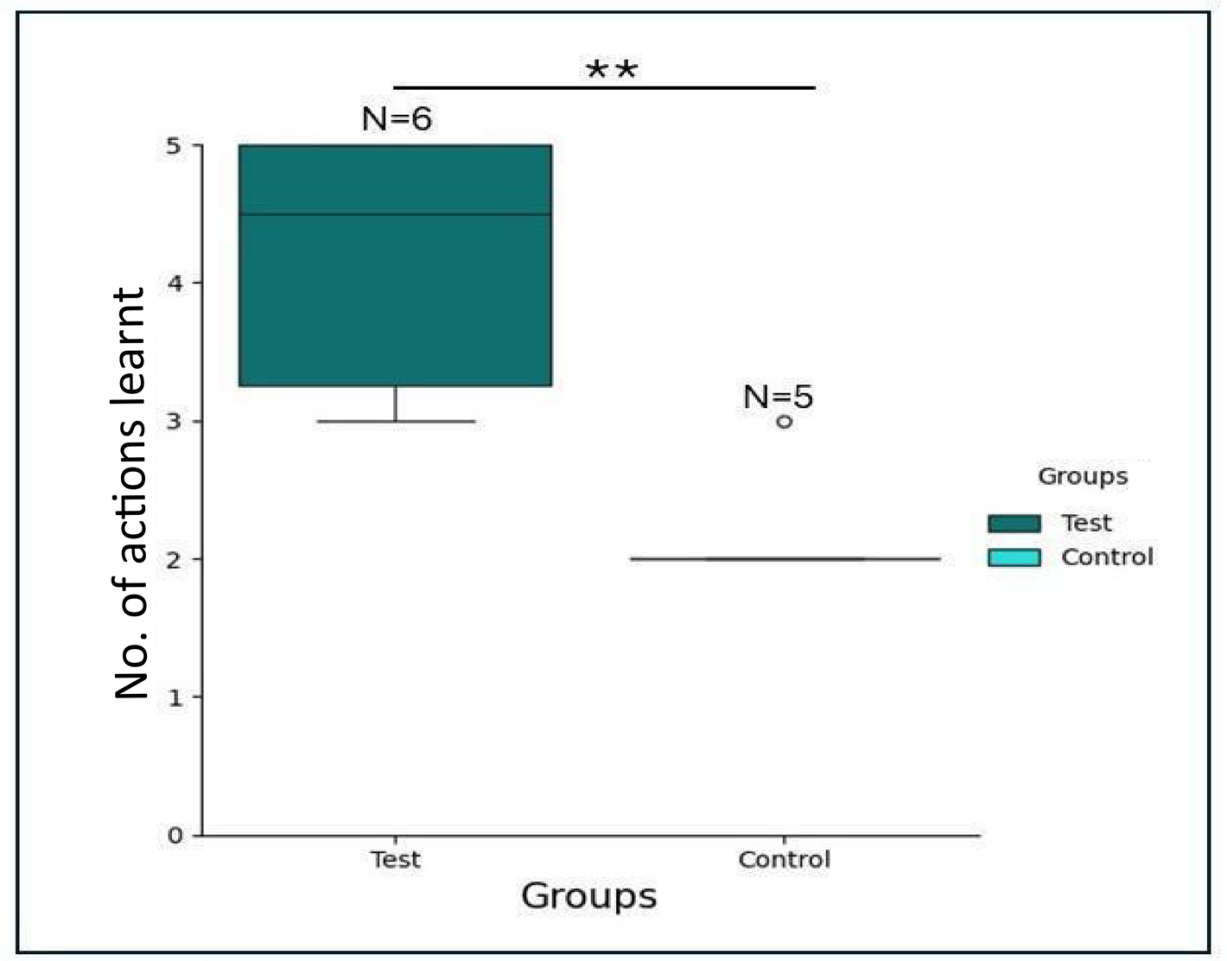
Number of target actions learnt by the subjects in the two experimental groups. The box plots show that the subjects in the test condition (teal blue) performed significantly better than the control group, denoted by asterisks (Wilcoxon-rank-sum-test (W = 25, *p* = 0.005). Error bars, mean ± SEM.

A total of 462 sessions, each consisting of 10 trials (462 × 10 trials = 4620 trials) were conducted with the two experimental groups to test for third-party imitation with the five target actions. The subjects in the test condition expressed significantly more target actions upon commands than subjects in the control condition (Wilcoxon-rank-sum-test: W = 25, *p* = 0.005; effect size of *r* = 0.933, 95% CI [0.794/∞]). A single, 7th subject from the test group did not learn any action and was removed from the analysis (see Supplementary information for details), reducing the sample size to 6 in the test condition. The 6 birds in the test group learnt on average 4 out of 5 actions (Mean = 4.16, SE = ± 0.4) whereas the control group learnt 2 (Mean = 2.20, SE = ± 0.2) as illustrated in Fig. 2. Table 1 gives an overview of the group performance while individual performance for both groups is provided in Supplementary Information Table S2.

**Table 1:** Number of subjects in the test and control conditions who learnt the behaviours.

Actions	Topology	Demonstrator	Test (*N* = 6 excluding a subject)	Control (*N* = 5)
Lift leg	Right (demonstrated)	Charlie	*N* = 5	*N* = 1
	Left		*N* = 1	*N* = 2
Spin	Clockwise		*N* = 4	*N* = 0
	Counterclockwise (demonstrated)	Gargamel	*N* = 2	*N* = 4
Fluff	Head	Charlie	*N* = 5	*N* = 3
Vocal	No distinction	Gargamel	*N* = 5	*N* = 1
Wings	No distinction	Charlie	*N* = 3	*N* = 0

### Response accuracy

**Fig. 3:**
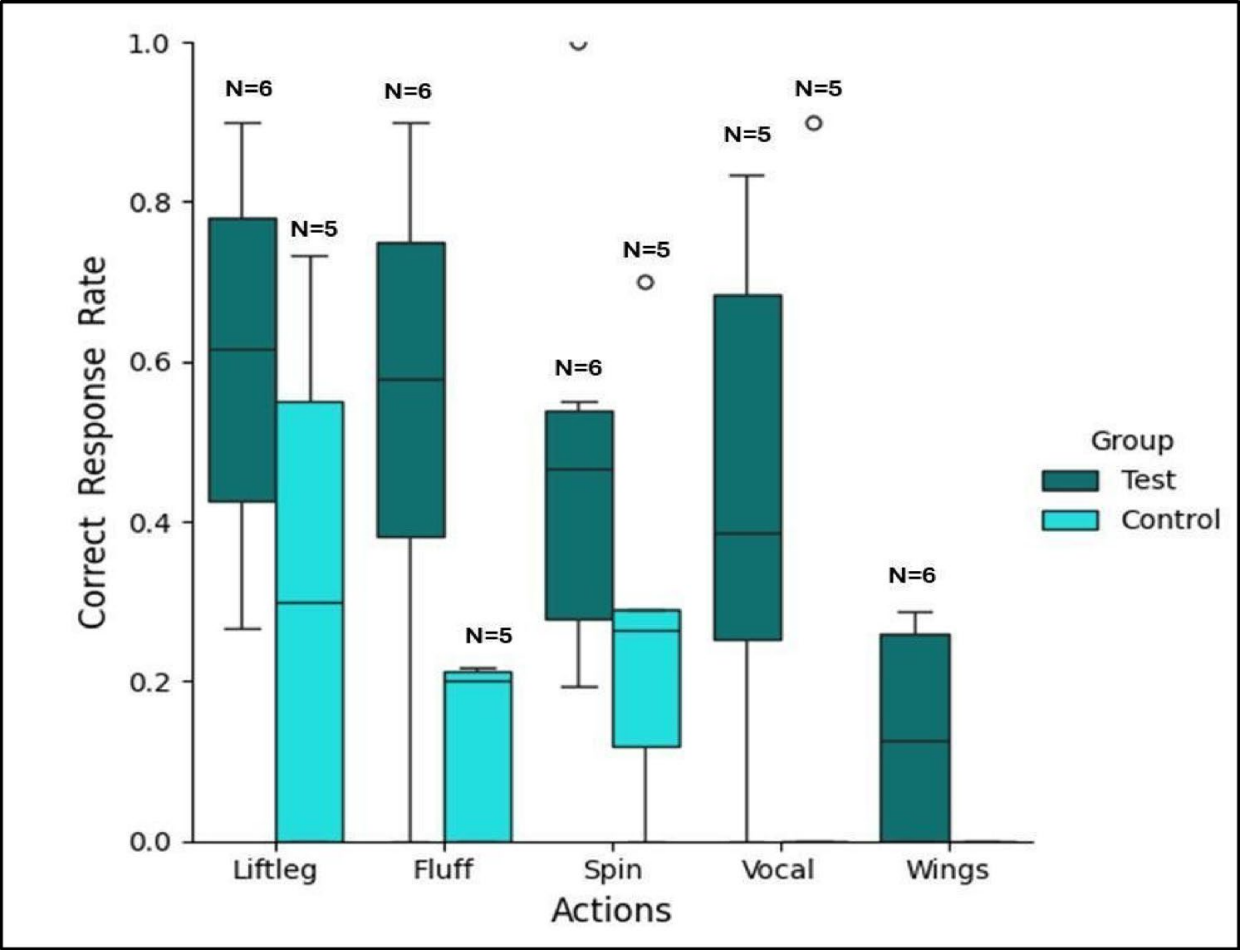
Mean correct response rate for each target action in the two experimental groups. The box plots show the mean response rate (y-axis) for each action (on the x-axis) for the test group (teal blue) and the control group (light blue). Error bars, mean ± SEM.

Mean correct response rate for each subject was calculated as the total number of target action responses (= correct responses) across the total number of trials required to reach the learning criterion (80% correct responses in two consecutive sessions) for each action. The fitted GLMM was highly significant with group Test (β = 0.25, t = 3.8, *p* = 0.0001, 95% C.I = [0.12, 0.38]) and action ‘flap wings’ (β = -0.27, t =-2.5, p = 0.02, 95% C.I = [-0.48, -0.06]) correctly predicting the response rate. Thus, the subjects in the test condition showed significantly higher mean correct responses (p = 0.0001) than the subjects in the control condition overall for the five target actions (see Fig. 3), while for the action *‘flap wings’* the response rate was the least. After the first action was learnt, subjects in both groups initially continued to perform this very action when experimenters switched to the next novel command in the consecutive following sessions (a carry-over effect). But the subjects in the test condition switched to performing the second action faster than the subjects in the control condition (who learnt only two actions), which can be attributed as an effect of demonstration in the test group (see Supplementary Information Figure S2).

### Learning speed

**Fig. 4:**
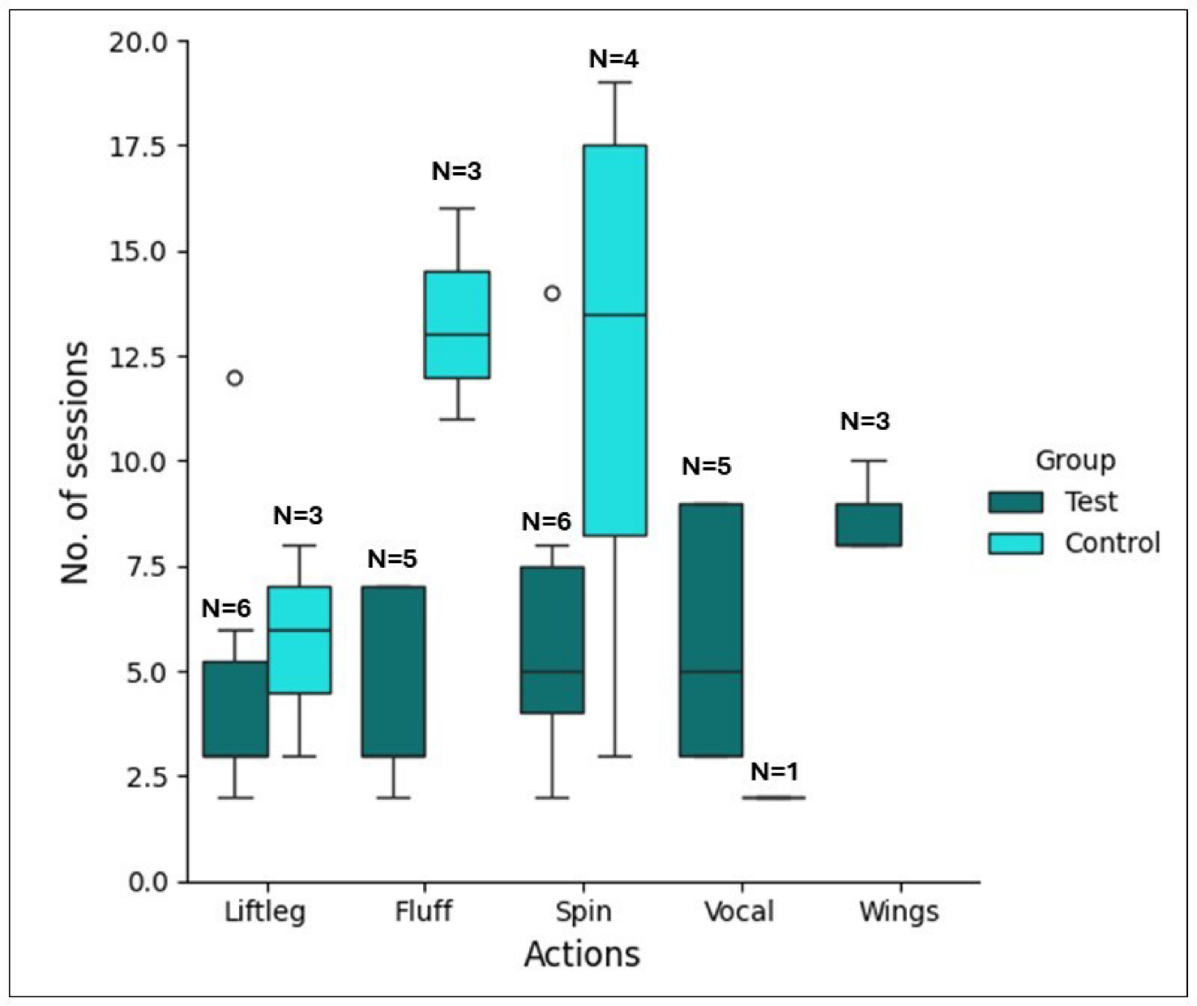
Mean number of sessions required to learn each target action. The box plots show the mean number of sessions (y-axis) required to learn each action (on the x-axis) for the test group (teal blue) and the control group (light blue). Error bars, mean ± SEM.

The total number of sessions required to express each target action was considered as the learning speed for that action. In accordance with our predictions, a GLMM fitted to predict the learning speed, revealed that the test group learned to express the target actions faster than the control group. The test group took significantly fewer number of sessions than the control group to learn to express the target actions upon command (GLMM: *N* = 11, β = -0.55, *z* = -2.51, *p* = 0.01, 95% C.I = [-0.95, -0.11]) overall). The random effect for subjects had near-zero variance, indicating minimal between-subject variability in intercepts. Figure 4 shows the mean learning speed of the two groups for each target action. Post-hoc analysis using a Wilcoxon-rank sum test showed that for the action *‘fluff’*, the five successful birds in the test group took significantly fewer sessions than the three successful birds in the control group (W = 15, *p* = 0.03) to learn to express this action. Such a significant difference was not observed for the other actions.

### Individual variation in action topography

#### Lift leg

The successful subjects lifted either of their feet and held it up in the air near the chest with four digits splayed out (see Table 2 for criterion). Although the majority of the test birds (see Table 1) lifted their ‘right’ foot as they reached the learning criterion, matching the demonstrator’s action, there was no significant difference in foot use preference between the groups (Fisher’s exact test: *p* = 0.22).

**Table 2:** Description of the target actions and the gestural commands used in the experiment.

Target actions	Topography of demonstrations	Gestural commands	Criteria for correct response
1. Lift leg	Demonstrator lifts the right foot up with splayed out digits and holds it in the air near the chest.	Experimenter holds the pointed index finger of her right arm at a chest level of the subject keeping an optimum distance from the subject.	Any foot can be lifted up and held in the air for some time, close to the chest, with digits splayed out (i.e. without grasping any object)
2. Spin	Demonstrator makes a 360 degree turn on the perch in an anticlockwise direction.	Experimenter lifts her right arm up, little above the eye level of the subject and uses two pointed fingers to make repeated anticlockwise round motions.	Subject has to complete 360 degree rotation on the perch in any direction (clock or anticlockwise).
3. Fluff	Demonstrator rapidly shakes the head.	Experimenter folds the three middle fingers and splays out the thumb and little finger of right arm. She holds the right arm up at the chest level of the subject and makes a to and fro motion.	Shaking of head is considered, not the whole body. In rare cases, the head shake was accompanied by body shake which was rewarded.
4. Vocal	Demonstrator gives an audible call from its natural vocal repertoire.	Experimenter makes a gesture with slightly folded right palm, opening and closing the fingers gently at the chest level of the subject.	Any vocalisation from the repertoire of the subject is accepted.
5. Flap Wings	Demonstrator flaps both wings with force while sitting on the perch.	Experimenter waves with her right palm in a to and from motion in front of the subject.	Flapping of both wings while sitting upright on a perch will be considered only. Flicking movement of wings usually associated with begging behaviour of juveniles will not be accepted.

#### Spin

A difference was observed between the test and the control group for the action spin. Four out of six (66%) individuals in the test group preferred to spin in clockwise direction - which was mirroring the direction of the demonstrator’s movement, i.e., rotating in the opposite direction. In contrast, the four control subjects who learnt the action, spun in the anticlockwise direction like the trained demonstrator did i.e., in the same direction as the circular movement of the experimenter’s forehand. Statistically, there was a trend (Fisher’s exact test- *p* = 0.07) in the number of subjects rotating in the clockwise vs. counterclockwise direction, showing a potential difference in the underlying mechanism of learning ‘*spin’* between the two groups (see Figure S1).

#### Fluff

*‘Fluff’* was characterized by a head shake with the feathers around the head and neck standing up as if aroused (see Table 2 for criterion). Rarely, the head shake would be accompanied by the body shake as observed in a few trials in one test bird (Iron man). We detected no other difference between the performance of the two groups in this action.

#### Vocal

As we considered any call or vocalisation as a correct target action for ‘vocal’, we did not analyse the spectrograms of the test subjects for similarity with the demonstrator or controls.

#### Flap wings

*‘Flap wings’* was the last action the subjects in the test group learned. Half of the test subjects, but none of the control subjects learnt the action *flap wings* confirming the improbability of the action to occur outside of demonstrations. One control subject repeatedly made characteristic species-typical begging movements with its wings accompanied by vocalisations. The topography of the begging movement did not match the target action (see Table 2 for criterion) and therefore was not rewarded. Another bird (in test condition) - the youngest (half-year-old) individual also showed begging movement with its wings. This was also considered an incorrect response and was not rewarded.

### Spontaneous imitation of target actions in the test group

In the test condition, a few cases of spontaneous imitation, which were not rewarded, occurred. In these cases, the subjects imitated the demonstrator in parallel to or shortly after the demonstration, i.e., within the 3–5 s gap before the hand command was given, prior to the first rewarded test trial (the production of the correct target action was only rewarded if it was produced in response to the hand command) of each action. Spontaneous imitation was observed with the actions *‘spin’* (all six test subjects produced *‘spin’* at least once, total events = 21, Mean = 3.5, S.E = ± 1.0), *‘fluff’* (five subjects produced spontaneous *‘fluff’* at least once, total events = 10, Mean = 1.67, S.E = ± 0.71) and *‘vocal’* (two subjects produced *‘vocal’* at least once, total events = 4, Mean = 0.8, S.E = ± 0.58). For the actions *‘lift leg*’ and *‘wing’*, no spontaneous imitation occurred prior to the first rewarded test trial.

### Serial order of target actions learnt

To test whether the subjects would learn to express certain action orders differently (order effect), we fitted a cumulative link model (CLM) with a logit link function to assess how the order of behaviours learnt was influenced by ‘action’ type and groups (test and control). Group: Test had a trend-level effect (*p* = 0.073). The action *‘lift leg’* was usually the first action to be learnt, though the result was not statistically significant (β = -1.67, z = -1.7, *p* = 0.08). *‘Flap wings’* was the last action (5th in order) to be learnt for all subjects in the test group (β = 4.0, z = 2.44, *p* = 0.01, 95% C. I= [1.1, 7.9]). No order effects were observed in the control group.

## Discussion

Untrained, non-enculturated blue-throated macaws – a non-domesticated animal species – imitated intransitive actions in a third-party setting, providing the first evidence for third-party imitation outside humans. The macaws learnt to produce five out of five rare intransitive actions (rarity established via baseline data) by passively observing the communicative interaction between a trained conspecific and a human experimenter as a third party. In contrast, the birds in the control condition managed to learn only two of those actions on average and one action was not learnt at all. The finding that the macaws learned to express more target actions in response to human hand commands as well as learning them faster and more accurately in this third-party setting than when they did not observe the third-party interaction, corroborates that the macaws in the test group imitated the actions of the conspecific demonstrator in response to the human experimenter. Our findings suggest that third-party imitation exists in macaws, and perhaps other parrot species, who may have evolved the ability to imitatively learn specific behavioural responses by observing the interactions of conspecifics without directly engaging with them, i.e., by third-party imitation.

The paradigm was demanding, considering that the parrots were not only required to imitate rare intransitive actions, but also learn about the correct interactive context between the conspecific and the experimenter in which the actions should be displayed (i.e., in response to specific, arbitrary human gestural commands), in order to obtain a reward. This is also evident from the final discrimination test, in which the test birds performed correct target actions well above chance level, when prompted with the hand commands in randomised order, without any aid of demonstrations (see results in Supplementary Information)— successfully distinguishing between the gestural commands and responding correctly in the interactive events. Previously, only dogs have been tested and failed in a comparable third-party setting in which a demonstrator dog responded to verbal human commands novel to the subject^5^. It is remarkable that dogs that (i) have life-long training of responding to human commands, (ii) exhibit a long history of domestication and (iii) can be trained to do motor imitation^20,57^ fail to imitate in a third-party setting spontaneously, whereas macaws, an undomesticated species, with naïve subjects that had never been trained to imitate or to perform any actions upon human instruction, succeeded. This could be attributed to the fact that the actions in Tennie et al.’s study^5^ like those in the present study, were intransitive. Domestication may have led dogs to become more responsive to transitive actions involving objects and clear goals^5^. However, to imitate intransitive actions, dogs need explicit training^20^. This may explain why they struggled to repeat species-typical behaviours that are not goal-directed, such as ‘lying down’ in response to an arbitrary vocal command. Parrots, which live in complex fission-fusion societies^46^ and regularly engage in conspecific social interactions with new group members or have to integrate into a newly formed group, may find arbitrary intransitive actions of conspecifics to be more salient stimuli for imitation. Perhaps as a result, they succeeded in ‘third-party imitation’ in the present study. Moore^10^ suggests that learning from third-party contexts requires an ability to recognise and imagine oneself in the third-party situation to correctly interpret a demonstration. This socio-cognitive skill is absent in one-year old human infants and develops from the second year of infancy^6,7^. Thus, learning via third-party imitation represents a significant evolutionary step that has previously not been reported in other animal taxa^5^. We cannot conclude from the present study whether macaws exhibit self-other equivalence and perspective-taking that are correlated to third-party imitation ability in children^7^ as testified by Mirror-Self-Recognition (MSR) tests. Mixed outcomes on MSR studies in different parrot species^58,59^ do not rule out the possibility of self-other distinction capacity in macaws. Nonetheless, our findings are not direct testimony for perspective-taking capacities in macaws and only imply that functionally, third-party imitation, although unreported and untested in (most) other non-human animals, may have evolved in this species, where learning social customs may be equally important as learning instrumental skills, to facilitate group cohesion.

In humans, learning social norms and traditions often require third-party imitation of community members in order to integrate into groups and participate in group events^11^. Most of these social conventions involve imitation of cognitively opaque arbitrary gestures and intransitive actions^60^. Blue-throated macaws live in complex social groups, like most parrot species, with fission-fusion dynamics^61^ which brings about frequent changes in group composition^62^. This necessitates faster group synchronisation and integration into new social groups. Third-party imitative learning of intransitive actions from conspecifics may facilitate group integration, affiliation and social bonding and may eventually give rise to cultural conventions of coordinated movements or possibly even gestures in parrots. Future studies will show whether this hypothesis holds just for macaws or for other parrot species.

Unlike other studies investigating intransitive action imitation in non-human animals (in DAID or DATOD studies; see above), the macaws had not received any active training to imitate or ‘copy’ on command, nor were ostensive signals used to direct the subjects’ attention to the interaction. We controlled for motivational factors like *social facilitation*^19^ by using a conspecific as a social facilitator in the control trials, which was passively present in the adjacent room in place of demonstrator. Vicarious reinforcement (subject observes the demonstrator getting rewards for performance)^63^ or observational conditioning (observer learns the relation between a stimulus and a reinforcer)^4^ may have played a role in motivating the test subjects to offer more actions that matched with the demonstrator’s after having observed a demonstrator receive rewards for performing actions^19^. However, correctly matching those demonstrated actions using the same effectors (body parts) in response to specific hand commands within a short period of time requires more than just motivational incentives. We ruled out goal emulation (copying the goal of the action) as all actions, except vocalization, were chosen to be goal-less, arbitrary motor movements. While vocal imitation is emulative^3^—since the observer can hear their own vocalization and adjust it to match the model’s without copying exact muscle movements—motor actions are not^2^. There are no salient goals in the chosen actions and thus goal copying (goal emulation) could be ruled out as the underlying social learning mechanism. The actions were not performed through motor mimicry or automatic imitation in test subjects, as they did not involuntarily replicate the action in the initial trials. They continued their previous actions (due to the carry-over effect, see Supplementary Figure S2) even after the experimenter switched to the new demonstration, making third-party imitation the more parsimonious explanation.

Against our predictions, the control subjects succeeded in acquiring the correct response to some of the commands (on average 2.2). A closer examination of what actions they learnt suggests that the birds did not just learn by trial-and-error. Two of the gestural commands may have involved some unintended cueing that led to the acquisition of those actions by the control group. For example, *‘spin’*, which was learnt by four control individuals seems to have been cued by the spin-like movement of the experimenter’s hand. The experimenter turned her two outstretched fingers in the anticlockwise direction which might have induced the control subjects to follow the finger movement and rotate in the same direction. The test subjects, however, rotated in the opposite direction to the movement of the fingers (see Supplementary Figure S1), thus copying the action of the demonstrator but in the opposite direction instead. This potential difference in learning mechanism lies in conceptualising ‘*spin*’ not as one, but a multi-step action that the test subjects copied from the demonstrator. This involves, starting from facing the experimenter, turning towards the window separating the rooms, facing opposite to the experimenter, then away from the other bird and lastly back to the experimenter (see Supplementary Figure S1). One may argue that rotating in the opposite direction to the model only indicates low-fidelity action matching. However, it has been shown in a DAID study that human children are prone to exhibit mirrored imitation that gradually decreases with age^64^. Thus, irrespective of the direction of rotation, copying the topography of the body movements throughout the action indicates action imitation, albeit with reduced fidelity.*‘Lift leg’*, which was acquired by three individuals in the control group (and all six individuals in the test group), may have been learnt via a potential correspondence of a lifted foot to the lifted index finger^65^ which constituted the gestural command. The remaining three commands however could not possibly have provided any potential cue towards the target action and were either not learnt at all by the control group (in the case of *‘flap wings’*) or by fewer subjects than in the test group (concerning ‘*fluff’* and *‘vocal’*). The action ‘flap wings’ was also learnt last in the order by the three test subjects who acquired it, implying the improbability of the behaviour as evidenced by the very low occurrence rate in the baseline observations. These actions linked with completely arbitrary hand commands learnt by the test group but mostly not learnt by the control group provide robust evidence for third-party imitation. The finding that some control subjects successfully produced correct responses to novel human gestures, even though they had never been trained to produce behaviours in interaction with a human experimenter, is very interesting in itself and suggests that the species has a very strong communicative tendency that even transcends species boundaries. Although the macaws in this study had minimal prior interaction with humans, the influence of captivity on their advanced communicative responsiveness towards humans cannot be entirely ruled out. Similar effects have been observed in captive chimpanzees, who exhibit referential pointing only in captivity—presumably due to human interactions—and rarely in the wild^66^.

For some actions (‘*spin’*,* ‘fluff’*,* ‘vocal’*) few instances of spontaneous imitation were observed in the test group concurrently with demonstrations before receiving the gestural command while for the other two actions (*lift leg* and *wings)*, none were observed. As there were only very few instances (*spin*: mean = 3.5, *fluff*: mean = 1.67, *vocal*: mean = 0.8) of these spontaneous imitations which also did not commence from the first trial of demonstrations, we ruled out contagious effect of any action^67^. Such instances occurred even without any prior reinforcement of that action. Those unrewarded imitation events show spontaneous, direct imitation and imply that parrots may have naturally evolved a tendency to imitate intransitive actions from conspecifics. In the large majority of cases however, the test group parrots clearly showed the action only in response to the gestural command, thus replicating the appropriate response witnessed as in a third-party context. The alternative explanation, that the parrots may not have learnt the appropriate response action from the third party setting but by imitating just the action of the conspecific model by second-party imitation, i.e., without paying any attention to the human gesture given just before, is unlikely. In such a case, we would have observed spontaneous imitations for the other two actions (*lift leg* and *wings*) too. However, in order to fully tease apart these two forms of imitation, future studies could incorporate additional control groups that systematically vary the presence of interactive cues and demonstration conditions. For example: (1) the observer sees only the demonstration, no cues given to demonstrator or observer; (2) the observer sees demonstration with cues but does not receive cues themselves; (3) the observer sees only the demonstration but receives cues themselves. Such a design would allow researchers to determine whether second-party imitation is equally driving the observed outcomes.

In this study, several limitations should be considered when interpreting the results. First, the sample size was relatively small as it is characteristic of many animal cognition studies working with species that are not easily available or kept in large numbers^68^. Second, due to the time-consuming training of the demonstrators, we decided to work with no more than five intransitive actions demonstrated in a third-party setting. Both points may limit the generalizability of our findings to the larger population. Future studies with larger sample size and more diverse behaviours could help confirm the findings. Additionally, the short duration of baseline observations limits the ability to draw firm conclusions regarding the relative improbability of the target behaviours. Hence extended longitudinal studies are recommended to further explore the causal relationship of probability of occurrence with imitation ability. However, as our task did not test for true imitation, we consider this limitation relatively unproblematic. Finally, although efforts were made to control for external variables, factors such as cueing from gestural commands may have played a role in the observed outcomes, particularly with regard to the control group, which learnt some actions contrary to our expectations. Future research should aim to control against possible unwanted cueing as part of the gestural commands more rigorously by using completely arbitrary hand gestures or completely arbitrary acoustic commands.

Overall, our study provides the first evidence for imitative learning from a third-party setting in a parrot, an ability previously recorded only in humans and, concurrently, the first evidence for third-party imitation of intransitive actions in a non-human species. Albeit a greater sample size would have been desirable and a third-party design involving an interaction between conspecifics would have been informative, the findings obtained from this study suggest that blue-throated macaws may learn imitatively by observing conspecific interactions. This may facilitate faster transmission of social information and group synchronisation that could provide adaptive benefits to a highly social species^69^. It also raises the possibility that macaws, and perhaps other parrots, may transmit and use gestures and bodily movements in the wild, maybe to a degree (not tested here) that may give rise to long-lasting cultures. Our study opens up a new line of inquiry into third-party imitation, encouraging future studies investigating interaction between two conspecifics being observed by a third party, and the imitation of intransitive actions in general, its evolution, and its potential importance for group cohesion and cultural evolution in a non-primate taxon: parrots.

## Materials and methods

### Subjects and housing conditions

Subjects were 14 (experimental subjects *N* = 12, demonstrators *N* = 2) hand-raised but early-on socialized and group-living captive blue-throated macaws (for details on rearing, age and sex refer to Supplementary Information and Table S2). They arrived at the Max Planck Comparative Cognition Research Station facility at Loro Parque Fundación, from a breeding facility of Loro Parque Fundacion, Tenerife, Spain, shortly before the commencement of the experiment. One control subject arrived later during the experiment and was not available for baseline observation. All subjects were group-housed in 3 adjacent semi-outdoor aviaries contiguous with the lab facility. All birds had *ad libitum* access to water and mineral blocks and were fed twice daily (for more details on housing and diet, please refer to Supplementary Information). Besides the two pre-assigned trained demonstrators, 12 subjects were divided into two groups (balancing for age and sex): (i) a test group that was tested in a third-party setting with demonstrator (*N* = 7) and (ii) a control group (*N* = 5) tested without a demonstrator in the first round and with a ‘social facilitator’ (as social enhancement control) in the second round (see below). The two demonstrators (8-year-old males) were behaviourally trained. They could perform 5 intransitive motor actions upon specific gestural commands (hand signals) from the experimenter (see Fig 1A). In contrast, the test and control subjects were completely naïve individuals that had not participated in any prior cognitive study previously and were only habituated to human presence, stay on a perch in the experimental room and receive food from human hands. They had not been trained to perform any behaviour on command or to copy actions of others. All subjects participated in the experiment voluntarily.

### Intransitive actions used in the experiment

Five arbitrarily chosen intransitive motor actions (all in the species repertoire), namely i) *‘lift leg’* ii) *‘spin’* iii) *‘fluff’* iv) *‘vocal’* and v*) ‘flap wings’* were used for the experiment. The detailed topography of each demonstrated action with the associated commands is described in Table 2. Additionally, the hand commands associated with the actions are illustrated in Fig. 1A. The two demonstrators performed the pre-assigned actions throughout the experiment: Charlie- *‘lift leg’*,* ‘fluff’* and *‘wings’*, Gargamel- *‘vocal’* and *‘spin’*, to control individual variation in the demonstrations. The order in which the five different actions and associated commands were introduced was randomized across subjects for both test and control groups (see Supplementary Table S1).

### Demonstrator training

The demonstrators had been trained to perform each of the five arbitrary intransitive actions in response to a specific hand signal as part of a previous study with the subjects^70^. Training involved operant conditioning and shaping methods^71,72^. The parrots learned to associate the sound of a whistle following a desired action with a food reward. The operant conditioning increased the likelihood of the occurrence of the target actions. Once the target actions had been reliably produced, they were associated with the respective commands (see details in Supplementary Information).

### Baseline: natural occurrence rate of the target actions

Behavioural observations were conducted throughout 16 days before the experiment commenced. We monitored each of the 11 subjects for 15 min (one control subject was not available as it arrived at the Max Planck facility later) per day for 16 days, resulting in 44 h of observation in total. During each focal sampling for continuous 15 min, it was noted if any of the five target behaviours were shown by each subject. The subjects were observed by the experimenter at different times of the day when they were not feeding, following some criteria (see Supplementary Information). The occurrences of each of those five actions from all the subjects in 44 h were summed to obtain cumulative scores (Supplementary Information). The rate of occurrence within a 12 s timeframe (the response time during experiment) was then calculated post-hoc as *Rate = (Cumulative score X 12)/44 × 60 × 60*. We set a post-hoc criteria for the rate of occurrence to infer which behaviours are more frequently observed in the natural repertoire- (i) Below 10%- infrequent, (ii) Below 5%- improbable and (iii) Below 1%- very improbable.

### Experimental setup

An illustration of the experimental set-up is shown in Fig. 1B. Testing took place in two adjacent indoor testing chambers, separated from each other with a transparent plexiglass window and equipped with lamps covering the birds’ full range of visible light (details in Supplementary Information), In each room, a standing perch (height: 1.13 m) was placed at a 1 m distance on either side of the plexiglass window. The demonstrator was placed on the perch to the left side of the glass while the observer was placed on the right side. Experimenter 1 stood in chamber 1 facing the demonstrator while experimenter 2 stood in the neighbouring chamber facing the subject. To ensure passive observation by the test subjects, both experimenters wore reflective sunglasses to avoid any unconscious cueing and remained passive throughout the test sessions and only interacted with the respective birds while giving the gestural commands and rewards. No other ostensive signals (calling or eye contact) were used. The setup for the control sessions was kept the same as the tests, with the control subject sitting on the perch in front of experimenter 2. The adjacent chamber was empty for the first round of testing in the control condition. From the second round, a conspecific bird (social facilitator)^19^ was placed on the perch accompanied by experimenter 1 – as a control for social enhancement^73^. We recorded the sessions on closed circuit television (cctv) cameras mounted on the walls of the experimental chambers, and a Sony HDR-CX240E video camera was additionally used. Testing took place from October 2021 to August 2022 between 10:30 and 14:00 regularly every week.

### General procedure

#### Reward contingency

The demonstrator was rewarded with a small piece of walnut for each correct demonstration in clear view of the subjects. No other actions of the demonstrator except the requested action during a trial was rewarded. The social facilitator in the control sessions was fed sunflower seeds every 2–3 min. The subjects in both conditions were rewarded with small pieces of walnuts for the target actions during the test trials. Any action that was not the target action, or that was performed outside of the 12 (± 2) sec interval (set a priori) following the gestural command given by experimenter 2 (i.e., the actual duration of the test trial, see below) was not rewarded. If a subject performed the matching action simultaneously with the demonstration or before it received its own gestural command, the subject was not rewarded to distinguish if any action was contagious that may be biologically predisposed to occur recurrently on observation even without rewards.

### Test condition

In each test session only one of the five actions was demonstrated and tested. At the beginning of each test session, the demonstrator and the subject were placed next to each other on the two standing perches. Both birds could clearly see both experimenters. Test sessions consisted of the 10 trials preceded by three demonstrations each, hence 30 demonstrations and 10 test trials in total. Experimenter 1 gave the gestural command requesting the demonstrator to perform a particular action (e.g., *‘lift leg’*)’. As soon as the demonstrator performed the action, she pressed a clicker and rewarded the bird with a small walnut piece in full view of the subject (for a description of the visual field of parrots see Demery et al. 2011)^74^. The three demonstrations were carried out in a row by experimenter 1 interacting with the demonstrator. Then, after a gap of 3–5 s (demonstrator completes eating the reward within this time), experimenter 2 started the test trial. She gave the same gestural command to the subject for 12 (± 2) seconds continuously, which experimenter 1 had given throughout the demonstrations (see Supplementary Videos S1-5). The timeframe was set arbitrarily to match the assumed attention span of a bird while giving a gestural command during one trial. If the subject performed the target action within this period of time, experimenter 2 pressed the clicker immediately and rewarded the subject with a walnut piece. If there was no response from the subject or a different action was performed, there was no reward, and experimenter 1 continued with the subsequent trial. The criterion for imitation (correct response) was the matching of the topography of model’s actions in response to the associated gestural commands. Factors like direction of movement or preference of foot use by the observer were excluded from the criterion (see Table 2).

#### Round 1 of testing

To avoid frustration of the subjects in case of continuous failure, we adopted a ‘rolling’ method for demonstration of the actions in two consecutive rounds. In the first round, the first action (as determined from the individually randomized order provided in supplementary Table S1) was demonstrated throughout a minimum of four sessions. In absence of any correct matching response from the subject within those four consecutive sessions, the 2nd action was demonstrated from the next session throughout at least four sessions, and the process continued for the following actions. If there was any correct response from the subject within those four sessions of a particular action, the testing continued until the subject reached the learning criterion of 80% correct responses (8 out of 10 correct trials) in two consecutive sessions. After all the 5 actions had been demonstrated in at least 4 sessions, round 2 commenced.

#### Round 2 of testing

Round 2 followed the same protocol as round 1, but now the minimum number of test sessions for an action was increased to 7 in a row. All actions that the subjects had failed to learn in round 1, were repeated in round 2 (see Supplementary Information Table S1). If a subject failed to respond correctly within those seven sessions, it was considered not to have learnt the action. If there was any target matching response within these seven sessions, the testing continued with that action until the subject had reached the 80% learning criterion in two consecutive sessions as in round 1.

### Control condition

The control sessions consisted of the same rolling method of two rounds employing the same number of sessions as the test condition. The first round was conducted without a conspecific present in the adjacent test chamber. However, in order to control for possible effects of social enhancement, a 2nd bird (social facilitator)^73^ was placed in the adjacent chamber from round 2 in the presence of experimenter 1. Experimenter 1 stood passively in front of the social facilitator, not giving any commands or cues. Occasionally, experimenter 1 would give sunflower seeds to the social facilitator to prevent it from begging using vocalisations or doing any other action. Experimenter 2 would give a hand signal to the subject for 12 (± 2) seconds continuously for the bird to respond (see supplementary videos S6-7). If the subject produced the target action, a clicker was pressed immediately followed by rewarding of the subject with a walnut piece, otherwise the next trial ensued after an inter-trial gap of 3–5 s. There were 10 trials in each session and the criteria for successful learning of a behaviour or failure were kept identical to the test condition. The social facilitator was rewarded only with sunflower seeds which was of lower value than walnut, to ensure the control subjects would not socially learn to remain passive like the social facilitator.

### Video analyses

The videos were independently coded by two experimenters (E.H and J.V who participated in the experiment) in equal ratio using Solomon Coder (version Beta 19.08.02, 2019 by András Péter). Production of target actions within 12 (± 2) sec after the hand signals in each session were coded as successful trials. Absence of any response or non-target responses were coded as failed trials. A Cohen’s κ = 0.96 for intercoder reliability was established through common coding of randomly selected 25 test and control sessions (5% of 462 sessions = 25 sessions or 250 trials) by both experimenters. Successful and unsuccessful trials were coded as a binary variable (success = 1, failure = 0), and we averaged it across total number of trials to get the response rate. We calculated the correct response rate as the measure for response accuracy. The total number of sessions faced in round 1 and round 2 to learn each behaviour was taken as the learning speed. For example, if a bird did not express any target action response in the four sessions of the1st round but learnt to express the target action in the 2nd round taking 13 sessions, we took the total session number as 17.

### Statistical analyses

All statistical analyses were performed in RStudio (R Core Team 2019, R version R.4.1.3). We conducted Wilcoxon-rank sum test to compare the number of actions learnt by the test and control subjects, due to non-normal distribution of the data. To determine the effect of experimental groups and the actions on mean response rate and learning speed of the birds, we ran a series of Generalized Linear Mixed Models (GLMMs) in the *lme4* package^75^ of R via *glmer* function using maximum likelihood estimation with Laplace approximation, including all explanatory variables (Fixed effects: groups, actions, groups*action interaction, random effects: individual identity) as part of exploratory analysis and selected the best model through backward selection. We fitted a Gaussian GLMM with identity-link function to predict ‘response rate’ and a negative binomial GLMM (Poisson GLMM showed overdispersion) with logit link function to predict learning speed or the ‘number of sessions’ required to reach the learning criterion. All model assumptions (i.e. normality of residuals, presence of outliers, dispersion) were checked using the DHARMa package^76^ in R, in addition to multicollinearity. The best fit models were selected on the basis of least AIC scores from the Akaike Information Criterion.‘Groups’ (test and control) and ‘actions’ (five levels: *lift leg*,* spin*,* fluff*,* vocal*,* wings)* were fitted as fixed effects and ‘individual identity’ was included as random effect to control for repeated measure in the final models. For the ‘number of sessions’ model, we excluded the data points on *wings* as only the test subjects were able to learn the action and thus fixed effect: ‘actions’ had only four levels. During model fitting, we encountered singularity issues despite specifying a relatively simple random effects structure (random intercept for subjects), indicating the estimated variance of random effects as (near) zero. Despite the singularity, we chose to present the results from the models with the full random effects structure to account for potential subject-level variability. To analyse the ‘order’ in which actions were learned, we initially fitted Cumulative Link Mixed Model (CLMM) from *ordinal* package^77^ to predict the ordinal response variable (order of action learnt) with ‘actions’ and ‘groups’ as fixed effects and ‘individual identity’ as random effect. However, this model produced a singular fit as well as several standard errors could not be computed, suggesting instability in parameter estimation. Hence, we opted to simplify the model by excluding the random effect of individuals and fitted a cumulative link model (CLM) instead, using *clm* function in the ‘*ordinal*’ package. The model assumption for an ordinal regression (proportional odds ratio) was checked using the *nominal_test* function, and we found no violation of assumption. We also conducted Fisher’s Exact tests of statistical significance to compare the topography of actions between the two groups.

## Electronic supplementary material

Below is the link to the electronic supplementary material.

Supplementary Material 1

Supplementary Material 2

Supplementary Material 3

Supplementary Material 4

Supplementary Material 5

Supplementary Material 6

Supplementary Material 7

Supplementary Material 8

## Data Availability

Raw and analysed data with R codes have been deposited at Figshare and is publicly available at DOI: https://doi.org/10.6084/m9.figshare.26819929.v3.

## Materials availability

Detailed description of the materials is listed in the Methods and Supplementary section. Any further information is available from the corresponding authors E.H (iesha.haldar@gmail.com) and A.M.P.B (auguste.bayern@bi.mpg.de).

## Code availability

This paper reports R codes for known statistical analysis like GLMM and Wilcoxon-rank sum tests.

## References

[1] Tennie, C., Call, J. & Tomasello, M. Ratcheting up the ratchet: on the evolution of cumulative culture. *Phil Trans. R Soc. B*. **364**, 2405–2415 (2009). 19620111 10.1098/rstb.2009.0052 PMC2865079

[2] Heyes, C. Imitation and culture: what gives? *Mind Lang.* **38**, 42–63 (2023).

[3] Byrne, R. W. & Russon, A. E. Learning by imitation: A hierarchical approach. *Behav. Brain Sci.* **21**, 667–684 (1998). 10097023 10.1017/s0140525x98001745

[4] Heyes, C. M. Social learning in animals: categories and mechanisms. *Biol. Rev.* **69**, 207–231 (1994). 8054445 10.1111/j.1469-185x.1994.tb01506.x

[5] Tennie, C. et al. Canis familiaris, fail to copy intransitive actions in third-party contextual imitation tasks. *Anim. Behav.* **77**, 1491–1499 (2009). Dogs.

[6] Matheson, H., Moore, C. & Akhtar, N. The development of social learning in interactive and observational contexts. *J. Exp. Child Psychol.* **114**, 161–172 (2013). 23164286 10.1016/j.jecp.2012.09.003

[7] Herold, K. H. & Akhtar, N. Imitative learning from a third-party interaction: relations with self-recognition and perspective taking. *J. Exp. Child Psychol.* **101**, 114–123 (2008). 18635193 10.1016/j.jecp.2008.05.004 PMC2577159

[8] Floor, P. & Akhtar, N. Can 18-Month-Old infants learn words by listening in on conversations?? *Infancy* **9**, 327–339 (2006). 33412677 10.1207/s15327078in0903_4

[9] Stenberg, G. Infant imitation in a third-party context. *IS* **21**, 387–411 (2020).

[10] Moore, C. Understanding self and others in the second year. in *Socioemotional Development in the Toddler Years: Transitions and Transformations* 43–65.

[11] Gaskins, S. & Paradise, R. Chapter five: Learning through observation in daily life. in *The anthropology of learning in childhood* 85–110 (2010).

[12] Lew-Levy, S., Lavi, N., Reckin, R., Cristóbal-Azkarate, J. & Ellis-Davies, K. How do Hunter-Gatherer children learn social and gender norms?? A Meta-Ethnographic review. *Cross-Cultural Res.* **52**, 213–255 (2018).

[13] Akins, C. K., Klein, E. D. & Zentall, T. R. Imitative learning in Japanese quail (Coturnix japonica) using the bidirectional control procedure. *Anim. Learn. Behav.* **30**, 275–281 (2002). 12391793 10.3758/bf03192836

[14] Zentall, T. R., Sutton, J. E. & Sherburne, L. M. True imitative learning in pigeons. *Psychol. Sci.* **7**, 343–346 (1996).

[15] Fawcett, T. W., Skinner, A. M. J. & Goldsmith, A. R. A test of imitative learning in starlings using a two-action method with an enhanced ghost control. *Anim. Behav.* **64**, 547–556 (2002).

[16] Voelkl, B. & Huber, L. True imitation in marmosets. *Anim. Behav.* **60**, 195–202 (2000). 10973721 10.1006/anbe.2000.1457

[17] Bates, L. A. & Byrne, R. W. Imitation: what animal imitation tells Us about animal cognition. *Wires Cogn. Sci.* **1**, 685–695 (2010). 10.1002/wcs.77 26271653

[18] Thorpe. Thorpe, W. H. *Learning and Instinct in Animals* 2nd edn (Harvard University Press, 1963).

[19] Zentall, T. R. Imitation: definitions, evidence, and mechanisms. *Anim. Cogn.* **9**, 335–353 (2006). 17024510 10.1007/s10071-006-0039-2

[20] Topál, J., Byrne, R. W., Miklósi, Á. & Csányi, V. Reproducing human actions and action sequences: do as I do! In a dog. *Anim. Cogn.* **9**, 355–367 (2006). 17024511 10.1007/s10071-006-0051-6

[21] Custance, D., Whiten, M. & Bard, A. (ed A, K.) Can young chimpanzees (Pan troglodytes) imitate arbitrary actions?? Hayes & Hayes (1952) revisited. *Behaviour* **132** 837–859 (1995).

[22] Call, J. Body imitation in an enculturated orangutan (*Pongo pygmaeus*). *Cybernetics Syst.* **32**, 97–119 (2001).

[23] Abramson, J. Z. et al. Contextual imitation of intransitive body actions in a Beluga Whale (Delphinapterus leucas): A do as other does study. *PLoS ONE*. **12**, e0178906 (2017). 28636677 10.1371/journal.pone.0178906 PMC5479519

[24] Abramson, J. Z., Hernández-Lloreda, V., Call, J. & Colmenares, F. Experimental evidence for action imitation in killer whales (*Orcinus orca*). *Anim. Cogn.* **16**, 11–22 (2013). 22875725 10.1007/s10071-012-0546-2

[25] Bauer, G. B. & Johnson, C. M. Trained motor imitation by bottlenose dolphins (*Tursiops Truncatus*). *Percept. Mot Skills*. **79**, 1307–1315 (1994). 7899015 10.2466/pms.1994.79.3.1307

[26] Tomasello, M., Call, J., Nagell, K., Olguin, R. & Carpenter, M. The learning and use of gestural signals by young chimpanzees: A trans-generational study. *Primates* **35**, 137–154 (1994).

[27] Tomasello, M. & Zuberbühler, K. Primate vocal and gestural communication. in The Cognitive Animal (eds Bekoff, M., Allen, C. & Burghardt, G. M.) 293–300. 10.7551/mitpress/1885.003.0041. (The MIT Press, 2002).

[28] Byrne, R. W. et al. Great ape gestures: intentional communication with a rich set of innate signals. *Anim. Cogn.* **20**, 755–769 (2017). 28502063 10.1007/s10071-017-1096-4 PMC5486474

[29] Tomasello, M. & Call, J. Thirty years of great ape gestures. *Anim. Cogn.* **22**, 461–469 (2019). 29468285 10.1007/s10071-018-1167-1 PMC6647417

[30] Halina, M., Rossano, F. & Tomasello, M. The ontogenetic ritualization of bonobo gestures. *Anim. Cogn.* **16**, 653–666 (2013). 23370783 10.1007/s10071-013-0601-7

[31] Tomasello, M. et al. The ontogeny of chimpanzee gestural signals: A comparison across groups and generations. *EOC* **1**, 223–259 (1997).

[32] Moore, B. R. Avian movement imitation and a new form of mimicry: tracing the evolution of a complex form of learning. *Behav* **122**, 231–263 (1992).

[33] Tennie, C., Call, J. & Tomasello, M. Untrained chimpanzees (Pan troglodytes schweinfurthii) fail to imitate novel actions. *PLoS ONE*. **7**, e41548 (2012). 22905102 10.1371/journal.pone.0041548 PMC3414512

[34] Todt, D. Social learning of vocal patterns and modes of their application in grey parrots (*Psittacus erithacus*)^1, 2, 3^. *Z. Für Tierpsychologie*. **39**, 178–188 (1975).

[35] Pepperberg, I. M. A review of the model/rival (M/R) technique for training interspecies communication and its use in behavioral research. *Animals* **11**, 2479 (2021). 34573445 10.3390/ani11092479 PMC8469950

[36] Wright, T. F. & Dahlin, C. R. Vocal dialects in parrots: patterns and processes of cultural evolution. *Emu - Austral Ornithol.* **118**, 50–66 (2018). 10.1080/01584197.2017.1379356 PMC6020700 29962561

[37] Zandberg, L., Lachlan, R. F., Lamoni, L. & Garland, E. C. Global cultural evolutionary model of humpback Whale song. *Phil Trans. R Soc. B*. **376**, 20200242 (2021). 34482732 10.1098/rstb.2020.0242 PMC8419575

[38] Badihi, G. et al. Dialects in leaf-clipping and other leaf-modifying gestures between neighbouring communities of East African chimpanzees. *Sci. Rep.* **13**, 147 (2023). 36604445 10.1038/s41598-022-25814-x PMC9814361

[39] Malherbe, M. et al. Signal traditions and cultural loss in chimpanzees. *Curr. Biol.* **35**, R87–R88 (2025). 39904312 10.1016/j.cub.2024.12.008

[40] Laland, K. & Janik, V. The animal cultures debate. *Trends Ecol. Evol.* **21**, 542–547 (2006). 16806574 10.1016/j.tree.2006.06.005

[41] Perry, S. Social traditions and social learning in capuchin monkeys (*Cebus*). *Phil Trans. R Soc. B*. **366**, 988–996 (2011). 21357221 10.1098/rstb.2010.0317 PMC3049088

[42] McGrew, W. C. & Tutin, C. E. G. Evidence for a social custom in wild chimpanzees?? *Man* **13**, 234 (1978).

[43] Silk, M. J., Croft, D. P., Tregenza, T. & Bearhop, S. The importance of fission–fusion social group dynamics in birds. *Ibis* **156**, 701–715 (2014).

[44] Langergraber, K. E. et al. The genetic signature of Sex-Biased migration in patrilocal chimpanzees and humans. *PLoS ONE*. **2**, e973 (2007). 17912352 10.1371/journal.pone.0000973 PMC1989134

[45] Goldsborough, Z., Webb, C. E., De Waal, F. B. M. & Van Leeuwen, E. J. C. Zoo-housed female chimpanzee adopts local female-specific tradition upon immigrating into a new group. *Behav* **158**, 547–564 (2021).

[46] Bradbury, J. W. 11. Vocal communication in wild parrots. in Animal Social Complexity (eds De Waal, F. B. M. & Tyack, P. L.) 293–316. 10.4159/harvard.9780674419131.c22. (Harvard University Press, 2003).

[47] Chakraborty, M. et al. Core and shell song systems unique to the Parrot brain. *PLoS ONE*. **10**, e0118496 (2015). 26107173 10.1371/journal.pone.0118496 PMC4479475

[48] Carouso-Peck, S., Goldstein, M. H. & Fitch, W. T. The many functions of vocal learning. *Phil Trans. R Soc. B*. **376**, 20200235 (2021). 34482721 10.1098/rstb.2020.0235 PMC8419581

[49] Benedict, L., Charles, A., Brockington, A. & Dahlin, C. R. A survey of vocal mimicry in companion parrots. *Sci. Rep.* **12**, 20271 (2022). 36470907 10.1038/s41598-022-24335-x PMC9722931

[50] Auersperg, A. M. I. et al. Social transmission of tool use and tool manufacture in goffin cockatoos (Cacatua goffini). *Proc. R Soc. B*. **281**, 20140972 (2014). 25185997 10.1098/rspb.2014.0972 PMC4173672

[51] O’Hara, M. et al. Wild goffin’s cockatoos flexibly manufacture and use tool sets. *Curr. Biol.* **31**, 4512–4520e6 (2021). 34469771 10.1016/j.cub.2021.08.009

[52] Klump, B. C. et al. Innovation and geographic spread of a complex foraging culture in an urban Parrot. *Science* **373**, 456–460 (2021). 34437121 10.1126/science.abe7808

[53] Bertin, A. et al. Facial display and blushing: means of visual communication in blue-and-yellow macaws (Ara Ararauna)? *PLoS ONE*. **13**, e0201762 (2018). 30133471 10.1371/journal.pone.0201762 PMC6104955

[54] Moura, L. N. et al. Gestural communication in a new world Parrot. *Behav. Process.* **105**, 46–48 (2014). 10.1016/j.beproc.2014.03.003 24631994

[55] Mui, R., Haselgrove, M., Pearce, J. & Heyes, C. Automatic imitation in budgerigars. *Proc. R Soc. B*. **275**, 2547–2553 (2008). 18664439 10.1098/rspb.2008.0566 PMC2605797

[56] Haldar, E., Subramanya, P. & von Bayern, A. M. P. Automatic imitation of intransitive actions in macaws. *iScience* **111514**10.1016/j.isci.2024.111514 (2024). 10.1016/j.isci.2024.111514 PMC11699809 39759005

[57] Fugazza, C., Petro, E., Miklósi, Á. & Pogány, Á. Social learning of goal-directed actions in dogs (Canis familiaris): imitation or emulation? *J. Comp. Psychol.* **133**, 244–251 (2019). 30407032 10.1037/com0000149

[58] Silveira, R. M. L., Almeida, J. M. & Alves, M. A. S. Rosy-faced lovebirds’, Agapornis roseicollis (Aves: Psittaciformes), response to their own image reveals self-recognition behaviour. *Behav* **160**, 889–909 (2023).

[59] Van Buuren, M., Auersperg, A., Gajdon, G. & Tebbich, S. Von bayern, A. No evidence of mirror self-recognition in Keas and goffin’s cockatoos. *Behav* **156**, 763–786 (2019).

[60] Gergely, G. & Csibra, G. Sylvia’s recipe: the role of imitation and pedagogy in the transmission of cultural knowledge. in Roots of Human Sociality (eds Enfield, N. J. & Levinson, S. C.) 229–255. 10.4324/9781003135517-11. (Routledge, 2020).

[61] Hesse, A. J. & Duffield, G. E. The status and conservation of the Blue-Throated Macaw *Ara glaucogularis*. *Bird. Conserv. Int.* **10**, 255–275 (2000).

[62] *Manual of Parrot Behavior*. (Wiley, 2006). 10.1002/9780470344651

[63] Bandura, A. Social learning theory. (General Learning press, 1977).

[64] Wapner, S. Imitation of a Model’s Hand Movements: Age Changes in Transposition of Left-Right Relations. *Child Development* (2025). 5687333

[65] Nehaniv, C. L. & Dautenhahn, K. The correspondence problem. in Imitation in Animals and Artifacts (eds Dautenhahn, K. & Nehaniv, C. L.) 41–62. 10.7551/mitpress/3676.003.0003. ((The MIT Press, 2002).

[66] Hobaiter, C., Leavens, D. A. & Byrne, R. W. Deictic gesturing in wild chimpanzees (Pan troglodytes)? Some possible cases. *J. Comp. Psychol.* **128**, 82–87 (2014). 24040760 10.1037/a0033757

[67] Tolman, C. W. & Wilson, G. F. Social feeding in domestic chicks. *Anim. Behav.* **13**, 134–142 (1965). 10.1016/0003-3472(65)90111-9 5882808

[68] Farrar, B. G. et al. Reporting and interpreting non-significant results in animal cognition research. *PeerJ* **11**, e14963 (2023). 36919170 10.7717/peerj.14963 PMC10008313

[69] Caldwell, C. & Whiten, A. Evolutionary perspectives on imitation: is a comparative psychology of social learning possible? *Anim. Cogn.* **5**, 193–208 (2002). 12461597 10.1007/s10071-002-0151-x

[70] Torres Ortiz, S., Smeele, S. Q., Champenois, J. & Von Bayern A. M. P. Memory for own actions in parrots. *Sci. Rep.* **12**, 20561 (2022). 36446997 10.1038/s41598-022-25199-x PMC9709151

[71] Pryor, K. & Ramirez, K. Modern Animal Training. in *The Wiley Blackwell Handbook of Operant and Classical Conditioning* (eds. McSweeney, F. K. & Murphy, E. S.) 453–482. 10.1002/9781118468135.ch18 (Wiley, 2014).

[72] Pryor, K. Reinforcement training as interspecies communication. in *Dolphin Cognition Behavior* 253–260 (2013).

[73] Franz, M. & J Matthews, L. Social enhancement can create adaptive, arbitrary and maladaptive cultural traditions. *Proc. R Soc. B*. **277**, 3363–3372 (2010). 20547762 10.1098/rspb.2010.0705 PMC2981925

[74] Demery, Z. P., Chappell, J. & Martin, G. R. Vision, touch and object manipulation in Senegal parrots *Poicephalus senegalus*. *Proc. R. Soc. B.* 278, 3687–3693 (2011). 10.1098/rspb.2011.0374 PMC3203496 21525059

[75] Bates, D., Maechler, M., Bolker, B. & Walker, S. lme4: Linear Mixed-Effects Models using ‘Eigen’ and S4. 1.1–37. 10.32614/CRAN.package.lme4 (2003).

[76] Hartig, F. & DHARMa Residual Diagnostics for Hierarchical (Multi-Level / Mixed) Regression Models. 0.4.7. 10.32614/CRAN.package.DHARMa (2016).

[77] Christensen, R. H. B. Ordinal Regression Models for Ordinal Data. 2023.12–4.1. 10.32614/CRAN.package.ordinal (2010).

