# Supplementary material for: Third-party imitation is not restricted to humans
Source: Sci Rep. 2025 Sep 4;15:30580. doi: 10.1038/s41598-025-11665-9 (PMC12411640; doi:10.1038/s41598-025-11665-9)
Supplement: Supplementary file 8 — Supplementary Material 8 [file 41598_2025_11665_MOESM8_ESM.pdf]

Supporting Information

Supplementary Methods

Rearing conditions of the subjects

At Loro Parque breeding facility, many parrot chicks (including all our subjects) are hand-raised every year by professionals. This involves the hand feeding with syringes in regular intervals without any further interaction, while the chicks are in artificial nests with conspecific chicks. However, as soon as they can perch, they are put together with conspecifics and have no human contact except for the handfeeding and as soon as they feed independently (ca 6 weeks old), they are socialized and group housed in large aviaries with conspecifics. The subjects were brought to the Max-Planck comparative cognition research station at Loro Parque Fundacion, Tenerife, Spain, shortly before the experiment commenced (see Table S2 for the ages of the different individuals). After they had got accustomed to their new environment and new home aviaries, they were observed throughout 16 days in those aviaries for the baseline data collection. In parallel the habituation to the experimental rooms and to the experimenters took place. Before the experiment commenced the subjects had been habituated to basic handling and clickers, remaining on the perch in the experimental chamber in the presence of a human experimenter and to receiving rewards from human hands. Otherwise, they had received no further training.

Housing conditions

All subjects were group-housed in 3 adjacent semi-outdoor aviaries contiguous with the lab facility. The aviaries measured approximately 1.80 × 3.40 × 3 m (width × length ×

height) with interconnected windows (1m x 1m) which remained closed throughout the experiment to avoid social conflicts between certain individuals. All birds had 24-hour access to the outside aviary, allowing them to follow a natural light cycle. Half of the aviary was outdoors so the birds were exposed to natural weather conditions. The other half was covered and lit with Arcadia Zoo Bars (Arcadia 54W freshwater Pro and Arcadia 54W D3 Reptile Lamp) that automatically followed the natural daylight regime. Outdoor temperatures during the research period fluctuated between 20 to 26 degrees Celsius during the day and 15 to 21 degrees Celsius during the night with interspersed periods of light raining. All birds had ad libitum access to water and mineral blocks. They were fed with fresh fruits, vegetables twice a day along with a seed mix only in the afternoon. Their daily nut ration was provided to them throughout the testing, training or enrichment sessions. To transport the subjects to the testing rooms, the aviaries were connected with mobile (1m x 1m x 1m) feeding cages which could be wheeled with ease with the birds inside. The birds were completely used to this procedure and entered the cages to be transported readily and voluntarily.

Training details

Note that 'lift leg' was trained predominantly using a target stick, by making the parrot grab the target with the right foot and then shaping it to only lift the foot without grabbing. For 'fluff', the experimenter sprinkled a few droplets of water on the head feathers of the bird to induce shaking of the head. 'Spin was trained by making the bird follow the target and consequently, the finger movement to complete a counter-clockwise rotation on the perch. 'Vocal' was scanned from their natural behaviours and opportunistically reinforced outside of the experimental chambers and associated with the command. 'Wings' required

intensive training. The experimenter held a perch in the air, with the bird sitting on it, She brought the perch down and up by a few inches several times which induced the bird to flap its wings to adjust its balance while sitting. The flapping on the hand-held perch was then transferred to the standing perch gradually.

Experimental setup

Testing took place in two adjacent indoor testing chambers, separated from each other with a transparent plexiglass window and equipped with lamps covering the birds' full range of visible light (Arcadia Zoo Bars). Measurement of each testing chamber was 2.5 m × 1.5 m × 1.5 m (height × width × length). The chambers were separated from each other with a transparent plexiglass window. of 1.0 m x 1.0 m with an opening in the lower part of the glass so that the parrots and the experimenters could clearly see and hear each other. The experiments could be observed by the Loro Parque zoo visitors through a one-way window such that the birds could not see or hear anything from outside the wall.

Final test for discrimination of gestural commands

To investigate whether the test subjects learned the correct context of producing the target actions, we tested them for discrimination between the gestural commands for the actions they learned without the aid of demonstrations, In two sessions, consisting of a maximum of 20 trials, with the randomized list of 3-5 actions learned by the subjects, the experimenter gave the gestural commands associated with the actions and waited for five seconds. If the subject produced the target action, the experimenter rewarded the subject with sunflower seeds and noted 1 for a successful trial during the live session. If the subject failed to produce the target action, or performed a different action within five seconds, the experimenter noted it as 0 for an unsuccessful trial, and the subject was unrewarded. If the

subject produced two actions simultaneously within 5 seconds, the trial was repeated. A third person, not involved in the experiment, coded 10% of the trials for interobserver reliability and a Cohen's kappa of > 0.9 was reached.

Statistical analysis for final test

We conducted two exact binomial tests to establish whether the test subjects produced the correct actions above chance level. For the first test we assumed that chance performance of each correct response of the three subjects (Marvel, IM and Carrot) who learned 5 behaviours as 1/5. For the second test with the other three subjects (Natasha, Thor, Pickle) who learned 3-4 behaviours, we assumed the chance level for performing each correct action as 1/3 (higher criterion). To account for multiple comparisons across the three subjects, Sidak adjustments were applied to the exact binomial tests, maintaining a family-wise alpha level of 0.05.

Supplementary Results

Baseline: Natural occurrence rate of the target actions

Except for self-defending from a conspecific attack, the action '*lift leg*' was not observed. Only transitive versions of *'lift leg'* which we did not count, were observed, such as scratching of head or eyes with the leg, tucking in of one leg while sleeping or resting and nibbling of the ring or digits while lifting the foot. Hence, '*lift leg'* as considered as the target response, never occurred. Defending from attack occurred 4x, scratching 84x, resting 86x, nibbling 52x. The most frequently occurring action that was recorded was expectedly *'vocal'* (657x), followed by *'fluff'* (68x). Flapping wings while sitting was observed 6x while full rotation on a perch as approximation to *'spin'* action was observed only once.

The rate of occurrence at 15 secs timeframe was calculated as *Rate=(Cumulative score X 12)/44 X 60 X 60*.

Number of actions learnt

A 7th test subject was removed from the analysis after she did not respond at al in the experiment. She had only recently been integrated into the group of our test birds at the age of 15 and did not socialize with the other individuals in the aviary for the entire run of the experiment, behaving passively throughout. We concluded that she had not yet sufficiently accommodated well to the group, the lab and testing contingencies. On these grounds, she was excluded from the analysis.

Final test for discrimination of gestures

Each of the test subjects performed the target actions well above chance level ($p<0.00001$) out of 40 trials in two sessions (20 trials each session) when tested through exact binomial tests.

#	ID	successes	trials	p_value	sidak_adjusted	significant
1	Marvel	37	40	7.672843e-20	0.000000e+00	TRUE
2	Carrot	30	40	1.061995e-13	3.187450e-13	TRUE
3	IM	29	40	1.172330e-12	3.516853e-12	TRUE

#	ID	successes	trials	p_value	sidak_adjusted	significant
1	Natasha	37	40	4.731293e-15	1.432188e-14	TRUE
2	Thor	36	40	9.017886e-14	2.704503e-13	TRUE
3	Pickle	33	40	1.620184e-10	4.860552e-10	TRUE

Effect of age

We tested whether age could affect the social learning ability in this species as well as explain the non-responsiveness of the single individual in the test group which was excluded from the previous analysis. To test the effect of age on the imitative learning ability of the individuals, we fitted a linear regression model with age predicting the number of actions learnt by the test group subjects by observing a conspecific. When we included the individual who did not learn any single behaviour in our model, the prediction was stronger with number of actions learnt significantly decreasing with age ($F_{(1,5)} = 8.188$, $\beta = -0.24$, R^2 of 0.545, $p = 0.035$). There was no difference between the young and the adults of the control group in their trial-and-error learning ($\beta = -0.04$, $p = 0.51$). **Figure S4** shows that young macaws are more prone to social learning of arbitrary actions from a conspecific demonstrator than the adults.

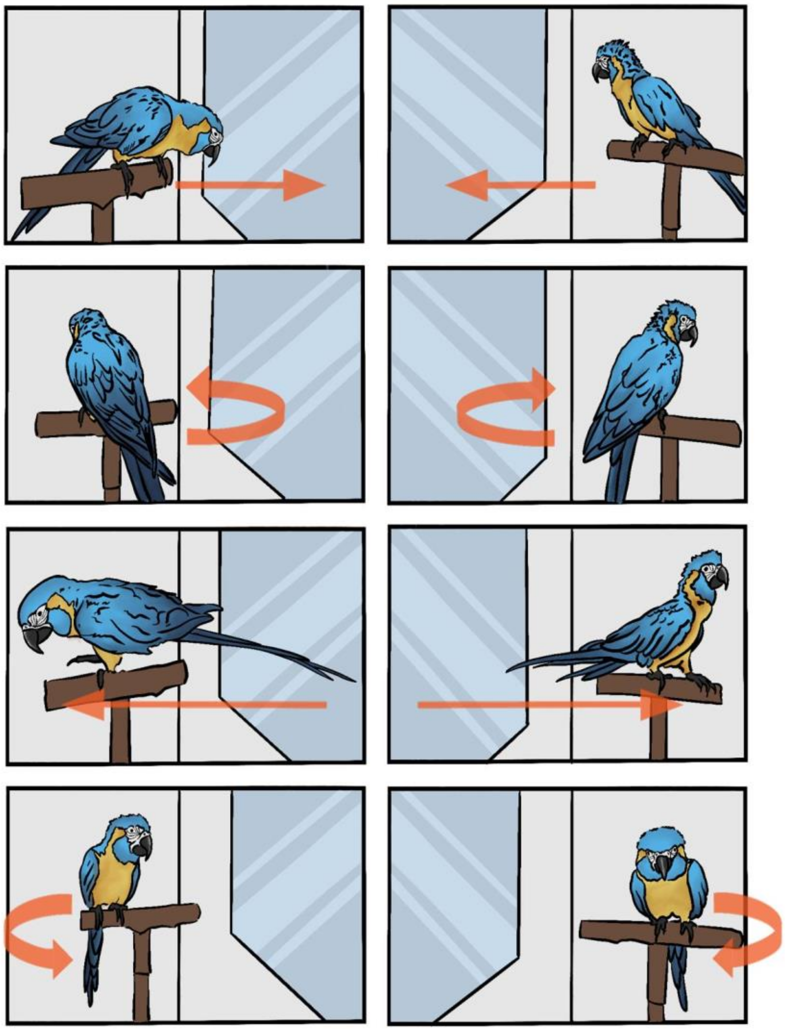

Fig. S1. ‘Spin’ target action of a test subject. The demonstrator is on the left panel and the test subject is on the right panel. The movement of the test subject during a ‘spin’ is shown in four consecutive steps.

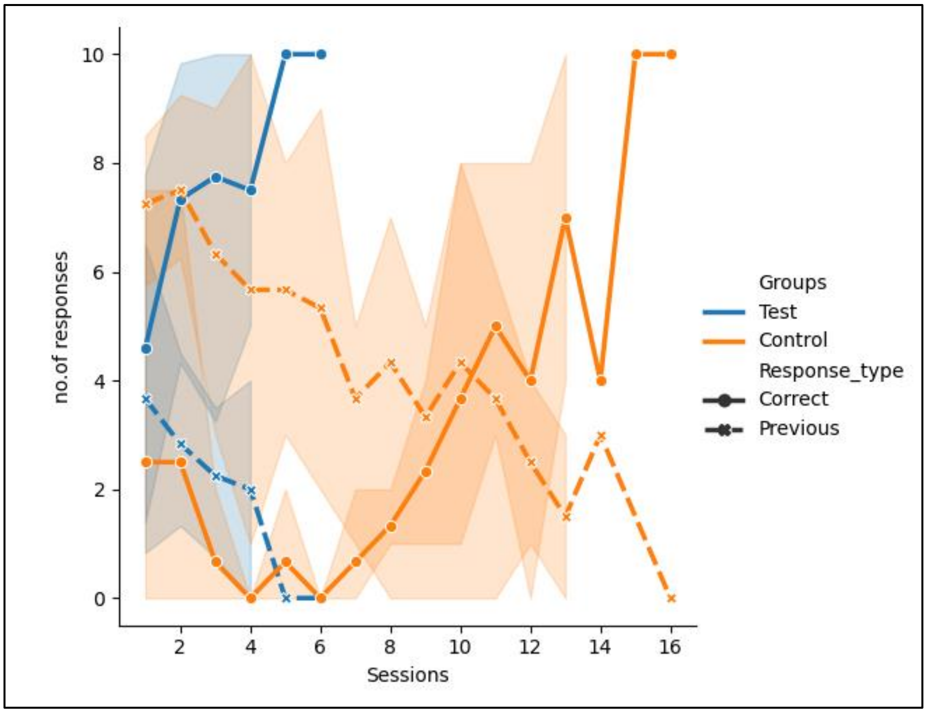

Fig. S2. Carry-over effect. Mean ‘correct responses’ of current action and mean of ‘previous action’ responses (first action learnt) are plotted across total number of sessions for the two experimental groups. At (0,0) subjects had learnt their preceding (first) action, followed by different response types (correct and previous) in the following sessions leading to learning of the current action denoted by correct responses above 80%. We did not consider the action switch for one control subject (Strange) who was intermittently absent from our research station and may have had some close human contact in the veterinary station. The subject had not produced any response in the first round of testing, but after the intermittent absence, it learnt two actions in the second round. This quick uptake of the behaviours may have been influenced by external factors, and hence we did not consider him for the action switching analysis.

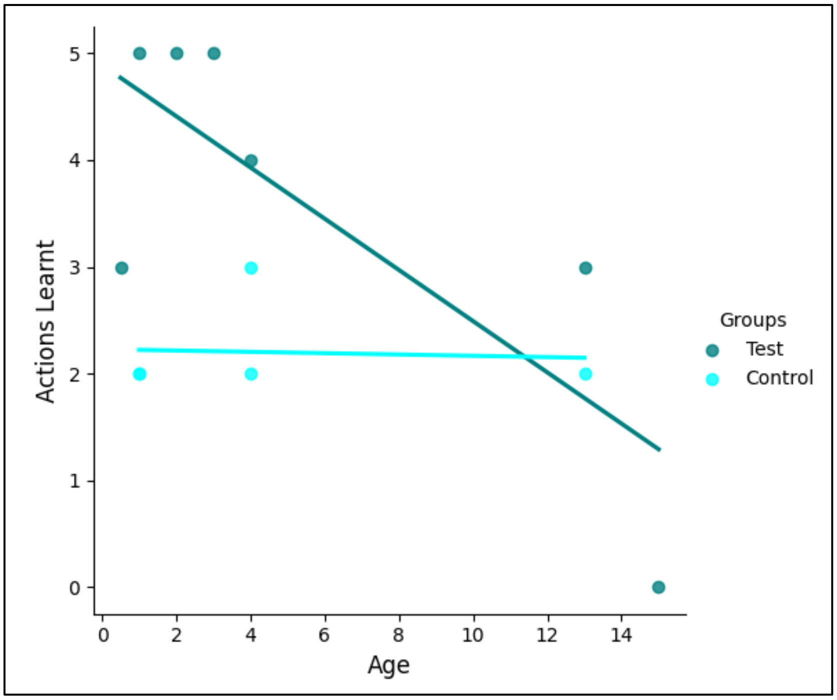

Fig. S3. **Scatter plot with regression lines showing the number of target actions learnt as function of age of macaws in the test (teal blue) and control (light blue) condition.** The number of actions learnt decreased significantly with age in the test condition when the subject (Wanda) who did not learn any action was included (linear regression, ($F_{1,5}$=8.188, β= -0.24, R^2 of 0.545, p= 0.035). The linear regression was not significant for the control condition ((β =-0.04, p =0.51).

Table S1. Details of the subjects in the two experimental groups, target actions learnt including their learning order and the learning speed (total sessions taken and round).

**Individual**	**Groups**	**Age (in years) / Sex (M/F)**	**Action demonstration order**	**Actions learnt (in order)**	**Total sessions taken (nth round)**	**Spontaneous imitations (actions learnt)**
Marvel	**Test**	2/F	Fluff	Lift leg	3 (1st)	0
			Spin	Vocal	3 (1st)	0
			Lift leg	Fluff	7 (2nd)	5
			Vocal	Spin	14 (2nd)	5
			Wings	Wings	8 (2nd)	0
Iron Man	**Test**	1/M	Fluff	Fluff	3 (1st)	1
			Lift leg	Lift leg	3 (1st)	0
			Wings	Spin	4 (1st)	7
			Spin	Vocal	3 (1st)	1
			Vocal	Wings	10 (2nd)	0
Carrot	**Test**	3/F	Lift leg	Lift leg	3 (1st)	0
			Fluff	Fluff	2 (1st)	1
			Wings	Vocal	5 (1st)	3
			Spin	Wings	8 (2nd)	0
			Vocal	Spin	8 (2nd)	5
Pickle	**Test**	0.5/M	Spin	Spin	2 (1st)	1
			Lift leg	Lift leg	2 (1st)	0
			Vocal	Vocal	9 (2nd)	0
			Fluff		11	
			Wings		11	
Natasha	**Test**	4/F	Lift leg	Lift leg	6 (1st)	0
			Spin	Spin	4 (1st)	1
			Vocal	Vocal	9 (1st)	1
			Wings	Fluff	7 (1st)	2
			Fluff		11	
Thor	**Test**	13/M	Fluff	Fluff	3 (1st)	1
			Lift leg	Spin	6 (1st)	2
			Wings	Lift leg	12 (2nd)	0
			Vocal		11	
			Spin		11	

Wanda (excluded)	**Test**	15/F	NA	NA	NA	
Morty	**Control**	4/M	Spin	Lift leg	8 (1st)	
			Wings	Spin	17 (2nd)	
			Vocal	Fluff	13 (2nd)	
			Lift leg		11	
			Fluff		11	
Rick	**Control**	4/M	Lift leg	Spin	3 (1st)	
			Spin	Fluff	16 (2nd)	
			Fluff		11	
			Vocal		11	
			Wings		11	
Pepper	**Control**	1/F	Lift leg	Spin	10 (1st)	
			Spin	Vocal	2 (1st)	
			Wings		11	
			Fluff		11	
			Vocal		11	
Sherlock	**Control**	13/M	Lift leg	Lift leg	3 (1st)	
			Fluff	Fluff	11 (2nd)	
			Spin		11	
			Vocal		11	
			Wings		11	
Strange	**Control**	1/M	Vocal	Spin	19 (2nd)	
			Fluff	Lift leg	6 (2nd)	
			Spin		11	
			Wings		11	
			Lift leg		11	

Video S1. Exemplary video of a *'spin'* test trial.

Three times the demonstrator responds to experimenter 1's hand command for *'spin'* by performing a *'spin'* followed by an immediate clicker and a reward. Following the three

reinforced demonstrations, the test group subject on the right side receives the same hand command by experimenter 2, which is continued until the subject responds correctly and receives a clicker and subsequent reward too.

Video S2. Exemplary video of a *'lift leg'* test trial.

Three times the demonstrator responds to experimenter 1's hand command for *'lift leg'* by performing a *'lift leg'* followed by an immediate clicker and a reward. Following the three reinforced demonstrations, the test group subject on the right side receives the same hand command by experimenter 2, which is continued until the subject responds correctly and receives a clicker and subsequent reward too.

Video S3. Exemplary video of a *'fluff'* test trial.

Three times the demonstrator responds to experimenter 1's hand command for *'fluff'* by performing a 'fluff' followed by an immediate clicker and a reward. Following the three reinforced demonstrations, the test group subject on the right side receives the same hand command by experimenter 2, which is continued until the subject responds correctly and receives a clicker and subsequent reward too.

Video S4. Exemplary video of a *'vocal'* test trial.

Three times the demonstrator responds to experimenter 1's hand command for *'vocal'* by performing a *'vocal'* followed by an immediate clicker and a reward. Following the three reinforced demonstrations, the test group subject on the right side receives the same hand command by experimenter 2, which is continued until the subject responds correctly and receives a clicker and subsequent reward too.

Video S5. Exemplary video of a *'flap wings'* test trial.

Three times the demonstrator responds to experimenter 1's hand command for *'flap wings'* by performing a *'flap wings'* followed by an immediate clicker and a reward. Following the three reinforced demonstrations, the test group subject on the right side receives the same hand command by experimenter 2, which is continued until the subject responds correctly and receives a clicker and subsequent reward too. The subject shows spontaneous imitation after it had been rewarded for the correct response.

Video S6. Exemplary video of a control trial *('vocal'*).

No conspecific demonstrator is present in the adjacent experimental room. The control group subject receives the hand command for '*vocal*', which is continuously repeated until the bird either shows the target action and is rewarded or 12sec have passed (shown here).

Video S7. Exemplary video of a control trial *('lift leg'*) with social facilitator present.

In round 2, experimenter 1 stands in front of a 'social facilitator' bird in the adjacent experimental room to the left, to control for social enhancement. The control group subject on the right receives the hand command for '*lift leg'*, which is continuously given until the bird either shows the target action and is rewarded or 12sec have passed (shown here).
